# Life cycle assessment and optimisation of surgical instrument trays for reverse shoulder arthroplasty

**DOI:** 10.1177/17585732251315424

**Published:** 2025-01-30

**Authors:** Isabella C Klarenbeek, Anne C van der Eijk, Esther RC Janssen, Freek Hollman, Paul C Willems, Okke Lambers Heerspink

**Affiliations:** 1 Department of Orthopedic Surgery, 8187 VieCuri Medical Centre, Venlo, The Netherlands; 2 Department of Orthopedics and Research School Caphri, Maastricht University Medical Centre+, Maastricht, The Netherlands; 3 Operating Room Department and Central Sterile Supply Department, Leiden University Medical Centre, Leiden, The Netherlands; 4 Department of Biomedical Engineering, 2860 Delft University of Technology, Delft, The Netherlands; 5 Radboud Institute for Health Sciences, IQ Health, 6034 Radboud University Medical Center, Nijmegen, the Netherlands; 6 School of Allied Health, 6032 HAN University of Applied Sciences, Nijmegen, the Netherlands

**Keywords:** Reverse shoulder arthroplasty, environmental impact, surgical instruments, life cycle assessment, sustainability, sterilisation

## Abstract

**Objectives:**

Shoulder arthroplasty has a large environmental impact. Part of the environmental impact is caused by the sterilisation of surgical instruments. This study examines the effect of optimising surgical instrument trays for reverse shoulder arthroplasty (RSA), to reduce the environmental impact.

**Methods:**

An adjusted LEAN 5s method was used to optimise the number of instruments of shoulder arthroplasty specific trays. A Life Cycle Assessment was performed to calculate the CO_2_-eq.

**Results:**

After careful selection, 139 of the 254 (55%) instruments were removed from the original RSA trays. Out of the 139 removed instruments, 19 were placed in a supplemental tray. The number of base trays was reduced with 3 trays. The estimated impact by reducing these trays from the standard pre-operative setup is a reduction of 28% of the environmental impact annually (524 kg CO_2_ equivalent).

**Discussion:**

This study confirms the feasibility of optimising instrument trays for RSA, offering a straightforward method to reduce the environmental impact of shoulder arthroplasty. Our results show that strategic instrument selection can contribute to lowering the environmental impact of orthopaedic surgery.

## Introduction

Given the substantial contribution of the healthcare sector to global warming, hospitals should prioritise minimisation of their environmental impact.^1–3^ The operation room (OR) is a resource intensive department and is a large contributor to the environmental impact of hospitals.^4–6^ Optimising processes within this department could significantly contribute to reducing this impact. The sterilisation of surgical instruments is one of the processes for the OR department that requires substantial energy consumption.^3,7,8^ One steam steriliser machine can use 54,000 kWh and 1.5 million litre water annually.^9^ After each surgery, all of the reusable surgical instruments need to be cleaned, disinfected and sterilised for the next surgery. A significant proportion of these instruments remain unused during surgical procedures.^10–12^ Because of changes in surgical technique, new surgical instruments are introduced and added to surgical instrument trays (SITs) over time. When new content is added to the SITs, current instruments are typically kept on the SITs, without consideration of their usefulness.^13^ It has been indicated in multiple studies that the removal of these unused instruments after careful consideration has no demonstrable impact on patient safety.^11,12,14–18^ Removing these unused instruments from the trays reduces the environmental impact, labour and sterilisation costs of the OR.^2,3,6–8,19^

Significant reductions have been observed for total-knee arthroplasty (TKA) and total-hip arthroplasty (THA) in 2 separate studies, with TKA showing reductions of 32.2% and 43.6%, and THA showing reductions of 17.5% and 41.1%, respectively.^16,17^ To our knowledge, no studies have examined the possibility of reduction of SITs for reverse shoulder arthroplasty (RSA) as yet. RSA, being a newer procedure that has gained broad acceptance and is still undergoing significant improvements, is expected to benefit even more from SIT optimisation.^20–22^ Given the recent innovations in RSA, we anticipate that the reductions in SIT for RSA will be similar to, if not greater than, those achieved in TKA and THA. Annually, 3000 RSAs are performed in the Netherlands, 5000 in the United Kingdom and 20,000 in the United States.^23–25^ Reducing the cleaning, disinfection and sterilisation of instruments and optimising surgical trays for RSA has the potential to substantially reduce the environmental impact of shoulder arthroplasty. Therefore, the aim of the present study is to reduce environmental impact of RSA by optimising surgical instrument trays.

## Methods

### Study design

This study was a single-centre observational study, performed in a large public teaching hospital. No ethical approval was required. The STROBE reporting guideline was used.^26^ Consensus based decision making rounds were used to decide how to optimise the SITs. A Life Cycle Assessment (LCA) of the cleaning, disinfecting, and sterilising of SITs was performed to show how much the environmental impact of RSA could be reduced. An explanation of terms and abbreviations can be found in Table 1.

**Table 1.** Terms and abbreviations.

*Terms* A **Life Cycle Assessment** (**LCA**) is a method to evaluate the environmental impact associated with a product, process, or service throughout its entire life cycle. A **functional unit** is the unit of analysis in a LCA. It is a standardised measure to quantify performance. It can be used as a reference point for comparing different products or systems in a LCA. According to ISO 14040/44, it is the specific quantity of a product or function that is analysed to ensure consistent and accurate comparisons. The functional unit defines the basis on which all data is normalised, allowing for an objective evaluation of environmental impacts. *Abbreviations* **OR **= Operation Room department **SIT **= Surgical Instrument Tray **RSC **= Rigid Sterilisation Container **CSSD **= Central Sterile Supply Department **RSA **= reverse shoulder arthroplasty

### Instrument trays

In this study, the RSA was performed using the universe reverse and modular glenoid system (Arthrex GmbH Munich, Germany). All ten of the SITs with instruments needed for implanting an RSA were included in the optimisation process. Our SITs differ from the international RSCs offered by our manufacturer in content and packaging. We use surgical instrument trays with fewer instruments as the standard international RSCs.

### Tray optimisation process

Tray optimisation was performed using consensus rounds in the five steps of the LEAN 5S^3,27^ approach:

1. *sort* (determine instrument usage)
2. *simplify* (remove unnecessary instruments)
3. *sweep* (confirm availability of needed instruments)
4. *standardise* (re-arrange and create a standard layout)
5. *success* (monitor success).

Step one to four of the LEAN process were performed during “consecutive consensus rounds”. Step five of the LEAN process was performed by using retrospective data on instrument use in elective reverse shoulder arthroplasty between April 3^rd^ 2020 and February 15^th^ 2024.

**Figure 1.**
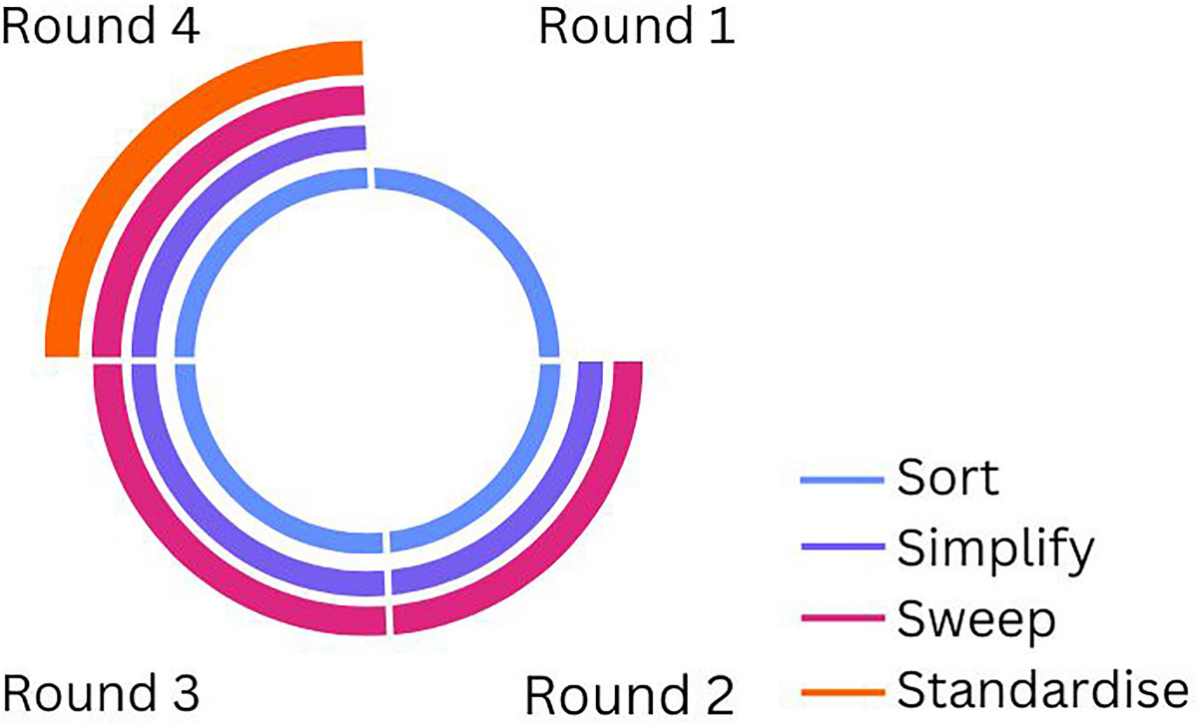
Overview of optimisation process steps: four rounds using step 1–4 of the LEAN 5S.

Four consensus rounds were performed, as outlined in Figure 1. The goal of these rounds was to achieve consensus on which instruments should be in the RSA base SITs and which instruments could be removed or transferred to the supplemental SITs. During the consensus rounds instruments that were categorised as ‘required’ were placed in one of the new base SITs. Instruments that were categorised as ‘supplemental’, were placed in additional SITs. The instruments that were no longer needed, were categorised as ‘removable’.

In the first round the researcher (ICK) and two orthopaedic surgeons (FH, OLH) specialised in shoulder surgery identified which SITs were to be used for this study. Relevant stakeholders were identified and invited to take part of the next consensus rounds by the researcher. A representative of the medical device company, the instrument tray manufacturer, an orthopaedic OR assistant, an expert from the Central Sterile Supply Department (CSSD) and the two orthopaedic shoulder surgeons. In each round, the researcher was involved as an independent party to provide critical feedback, ensuring that assumptions were challenged and discussed openly. The expert from the medical device company made the first proposal for instrument reduction based on the identified SITs. During the second round, the orthopaedic surgeons and orthopaedic OR assistant made corrections to this proposal under guidance of the researcher, (i.e., “required”, “supplemental” or “removable”) by considering the instrument usage during RSA for each individual instrument (LEAN steps sort, simplify & sweep). These recommendations were used as input for the third round. During the third round, the individual recommendations from the second round were again reviewed and the LEAN step sort and sweep were repeated collectively, during a consensus meeting involving the previous mentioned stakeholders. The result was a consensus based proposal for instrument reduction. This proposal was used for the fourth round, which again was a consensus meeting in which the instrument use of each instrument was again discussed until consensus was reached (LEAN steps sort and simplify). This time, physical SITs were used in the discussion. Availability of all instruments and standardised setups within the tray were discussed (LEAN steps sweep & standardise). Where the focus in previous meetings was solely on the content per tray, this meeting also included a comparison between trays. The instruments were moved between SITs to find the preferred layout, until consensus about this was reached to.

### Instrument sterilisation process

**Figure 2.**
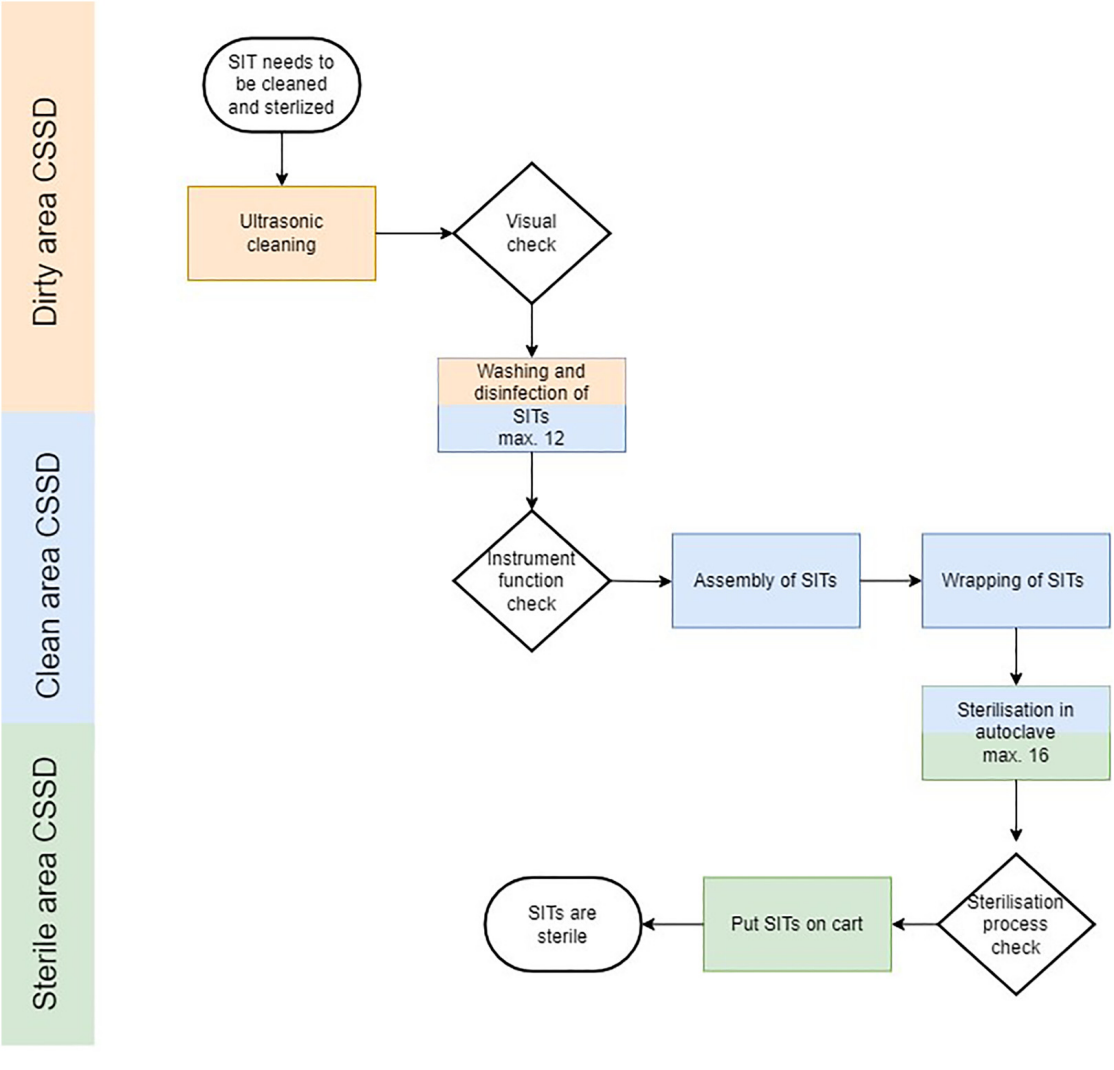
Process flow chart of instrument sterilisation process. The CSSD is organised into three distinct rooms, each dedicated to specific stages of the sterilisation process. The thermal disinfectors and steam sterilisers are strategically built into the walls separating these rooms to facilitate the smooth and efficient transfer of instruments. CSSD: Central Sterile Supply Department. SITs: Surgical Instrument Trays.

The process to clean, disinfect and sterilise the SITs has multiple steps and can be different per hospital. The SITs are cleaned, disinfected and sterilised in the Central Sterile Supply Department (CSSD) (see Figure 2). Instruments are put in an ultrasonic bath in our hospital (Elmasonic Generator MF-1000, Singen Germany), pre-cleaned manually, cleaned, disinfected and sterilised with cold and hot demineralized water with thermal disinfectors (Miele PG8528 D, Gütersloh Germany). After cleaning, disinfecting, sterilisation and drying, the instruments are placed back into position in the SITs and weighed, ensuring consistency. Instruments are arranged according to a picture that illustrates the placement for each instrument, maintaining uniformity in organisation for every tray. Finally, the trays are wrapped in two layers of Meatex 5 layers SSMMSS polypropylene wrap (Interster, Wormerveer the Netherlands) and sterilised in the steam sterilisers (Miele PS5662v, Gütersloh Germany) before they are transported to the OR.

### Life cycle assessment

The LCA was calculated for one cleaning, disinfecting, packaging and sterilisation cycle of one SIT (= ‘functional unit’). The calculation included the water, gas and electricity used by the thermal disinfector and steam steriliser and the transport, material and disposal of the polypropylene wrap and paper. The production and disposal of the machines and SIT was excluded from the LCA. Data on electricity, water, and detergent usage of the cleaning machine were gathered from the manufacturer. The conversion factors (kg CO_2_-eq.) were derived from Sustainability Impact Metrics Foundation^28^ and for soap the study Villota-Paz et al. was used.^29^ We used the indicator provided for drinking water in Europe to represent all types of water, as the database lacks specific indicators for other water categories. We anticipated that the impact of this simplification would be minimal.

Using this approach, we calculated the environmental impact in kg CO_2_-eq. for cleaning, disinfecting, packaging and sterilisation of one SIT, the original situation, the removed SITS and the optimised situation:

$$\mathrm{Total}\;\mathrm{kg}\;{\mathrm{CO}}_{2}-\mathrm{eq}.={\mathrm{CO}}_{2}-\mathrm{eq}.\;\mathrm{per}\;\mathrm{tray}*\mathrm{number}\;\mathrm{of}\;\mathrm{trays}$$

The reduction in kg CO_2_-eq. per year related to the optimisation was calculated using:

$$\begin{array}{cc} \text{Total}\;\text{reduction}\;\text{kg}\;\text{C}{\text{O}}_{2}-\text{eq}.\text{per}\;\text{year}= & \text{C}{\text{O}}_{2}-\text{eq}.\text{per}\;\text{tray}*\text{number}\;\text{of}\;\text{trays}\\ & *100\;\text{procedures}\;\text{per}\;\text{year}+\text{C}{\text{O}}_{2}-\text{eq}.\text{per}\;\text{tray}\\ & *\text{percentage}\;\text{of}\;\text{cases}\;\text{the}\;\text{supplemental}\;\text{tray}\;\text{has}\;\text{to}\;\text{be}\;\text{opened}\\ & *100\;\text{procedures}\;\text{per}\;\text{year} \end{array}$$

### Scenario analysis

The energy mix of the LCA was based on the electricity mix of our electricity supplier (wind energy) and the average for natural gas consumption of the Netherlands. A scenario analysis was performed to show the possible outcomes if a different energy mix was used. This included the average energy mix of The Netherlands, United Kingdom, Europe and United States. CO2-eq. indicators for electricity and gas were derived from the Sustainability Impact Metrics Foundation,^28^ the United States Energy Information Administration^30^ and the United Kingdom's Department for Environment, Food & Rural Affairs.^31^

## Results

Ten SITs were identified, including 254 surgical instruments. Discussion was held until consensus was reached. Of the 254 instruments, 139 instruments were classified as removable (see Table 2). After removal the overall reduction of instruments was 55%. After creating a new standardised arrangement of the instruments, 115 instruments were divided over seven SITs, making the removal of three of the ten SITs possible.

**Table 2.** Overview of the results. Of the total instruments, 115 instruments were required for reverse shoulder arthroplasty. Of the 139 instruments, 19 instruments were placed in the supplemental set.

Total instruments	254
Required	115
Removable	139
Supplemental	19

### Environmental impact analysis

By optimising the SITs, a reduction of three of the ten base trays was possible. The estimated environmental impact of one SIT was 1.87 (see Table 3 and supplemental file). The environmental impact of the ten SITs before the optimisation was 19.97 per procedure. The reducing of three trays saves 5.62 kg CO_2_-eq. The supplemental tray would need to be opened for 6.67% of the surgeries, because of bigger sizes. Annually, the optimisation could potentially save 524.34 kg CO_2_-eq. in our hospital. The overall reduction will be 28% because of this.

**Table 3.** Carbon dioxide equivalents emissions (kg CO_2_-eq.) per surgical instrument tray (SIT).

	Ultrasound	Washer- disinfector	Steam steriliser	Packaging	Total
1 SITs	0.01	0.62	0.82	0.42	1.87

**Figure 3.**
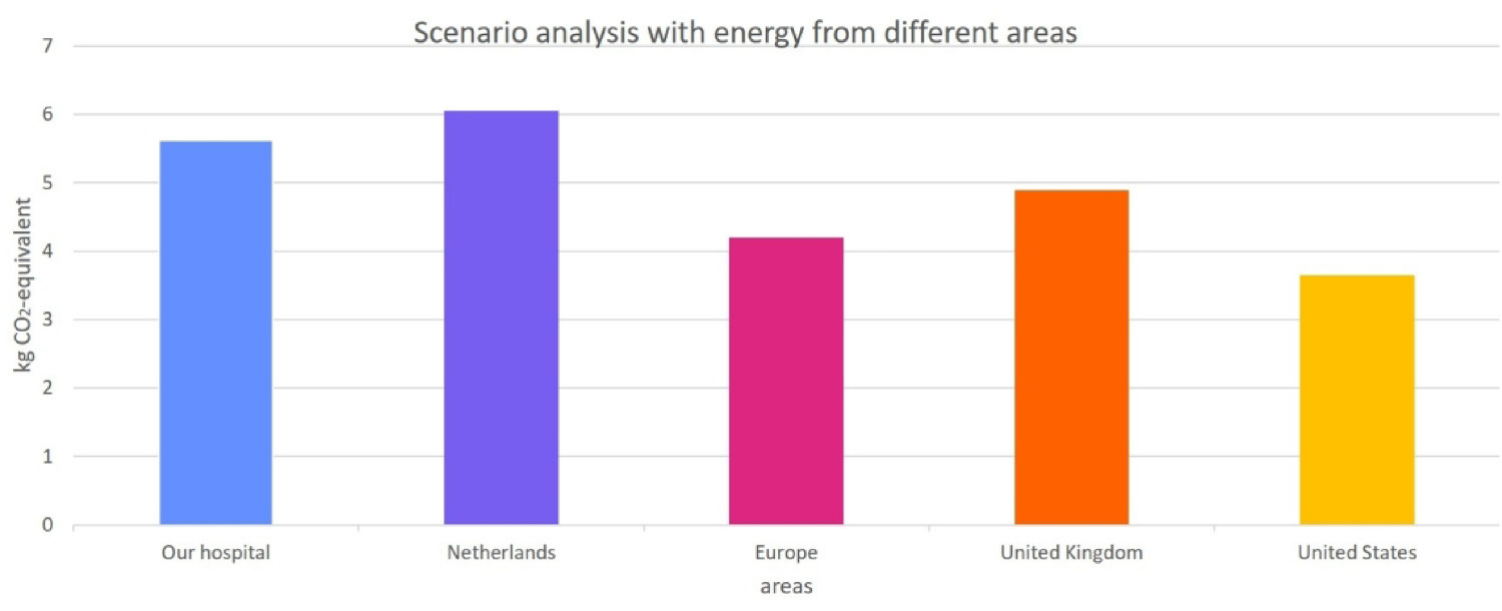
Scenario analysis of the effect of using different environmental impacts for energy. The outcome using our energy mix and the average energy mix of The Netherlands, United Kingdom, Europe and United States was calculated. The y-axis shows the potential difference of kg CO2-eq. of cleaning, disinfecting, and three 3 surgical instrument trays. Information from the Sustainability Impact Metrics Foundation (28), the United States Energy Information Administration (31) and the United Kingdom's Department for Environment, Food & Rural Affairs (32) was used.

A scenario analysis was conducted to evaluate various energy mix impacts, comparing the potential CO_2_-eq. savings from reducing three SITs across our hospital's energy mix, as well as the average energy mixes of the Netherlands, the United Kingdom, Europe, and the United States. The analysis revealed that the Netherlands average energy mix offers the highest potential CO_2_-eq. savings among the scenarios (see Figure 3). The estimated environmental impact of consumed gas was lower in the UK and US compared to the other areas. For electricity the CO_2_-eq. indicator was lower for our hospital (wind energy) and the UK. This influenced the results. Based on multiplying the outcome of the scenario analysis with the number of RSAs per year, the potential savings are 18,150 kg CO_2_-eq. in the Netherlands, 24,424 in the United Kingdom and 73,096 in the US.

## Discussion

We aimed to reduce the environmental impact of RSA by optimising SITs. In this study we achieved a reduction of 139 of 273 (55%) instruments and three SITs (30%) for RSA. The percentage reduction in instruments is greater than that of SITs because we opted for a layout with fewer instruments per SIT. This allows for more space between instruments when placed in the washer-disinfector and steam steriliser, improving cleaning and sterilisation efficiency. Besides lowering the environmental impact, reducing the number of SITs also lowers costs and labour.^3,7,8,10^ It can reduce cleaning and instrumentation setup in the CSSD and preoperative preparations and operating time in the OR. This time can be used for other activities. Another advantage might be an increase in work satisfaction, because CSSD personnel sees less unused instruments.^32^

The reduction of 55% of the instruments in our RSA SITs is higher than previous studies achieved^11,14–17^ that optimised surgical trays in other types of arthroplasty. Toor et al.^15^ have compared three methods (mathematical, clinical review, hybrid) to optimise the major orthopaedic tray, which showed a reduction between 23 and 47%. Belhouari et al. also compared the same three methods while optimising trays for spine surgery.^14^ Results were between 35 and 41%. The lowest results of both studies were of the clinical reviews, which were individual interviews with surgeons and nurses. Addition to the individual proposals, which stimulated idea sharing and a thorough evaluation of options. Additionally, the multiple review rounds we implemented also contributed to this outcome. The difference between the first concept version and the definitive version was 58 less instruments, an extra 23%.

### LCA results

Other studies have estimated the environmental impact from cleaning, disinfecting and sterilising one SIT and its packaging.^7,19^ The environmental impact of decontamination was 1.531 and the packaging was 0.387 kg CO_2_-eq. per SIT according to Rizan et al. The study of Schmidt et al. showed a total of 2.14 kg CO_2_-eq. for all life cycles of one SIT. Our study has small differences, which can be explained by the used carbon footprint database, differences in machines and system boundaries. We did not include the environmental impact of the production of the surgical instruments, because this is relatively small.^19^

### Strengths and limitations

One notable strength of this study was the comprehensive identification and involvement of all stakeholders. Engaging stakeholders early in the process helps to prevent potential issues for implementation. Involving the expert from the CSSD during LEAN 5S step 4 (standardisation) allowed them to provide crucial recommendations while the new standardised set-up was decided. This approach prevented the need for future changes related to sterilisation recommendations.

A limitation of this study was the use of retrospective data to assess the impact of reduction of instruments, LEAN step 5. Another limitation is the generalisation of the results. Other hospitals may differ in content of the SITs or have RSCs and the stakeholders might have different preferences, especially the orthopaedic surgeons who have used certain methods of their preferences. Differences in sterilisation protocols, might result in higher or lower savings of carbon emissions.

We did not directly measure water and energy consumption for the LCA; instead, we calculated these based on the manufacturer's information. We believe this will not significantly impact the final results. To address the impact of our energy mix, we conducted a scenario analysis to provide a more contextual perspective. This analysis showed that the origin of the electricity and gas influences the CO_2_-eq. indicators and thus the results of the environmental impact of removing a number of SITs.

### Implications for clinical practice

Our method using LEAN 5S is a new method to optimise SITs. We recommend when optimising SITs to involve all stakeholders in the process. This should at least include the surgeons, one OR assistant and an expert from the CSSD. We recommend appointing an independent process supervisor- someone without prior knowledge of the surgical instruments’ specific uses. In our case, the researcher (ICK) fulfilled this process supervisory role. An independent perspective allows to provide critical feedback, ensuring that assumptions are challenged and discussed openly. We recommend that future additions to the SITs are an excellent opportunity for optimisation of all operative procedures.

The retrospective check revealed that for 6.67% of RSA, a deviant baseplate was used. The commonly used baseplate was not in stock because of supply problems. These supply disruptions have the potential to necessitate additional instrumentation, potentially requiring the use of extra temporary SITs in the future.

Our study emphasises that it is possible to optimise SIT by improving sustainability of healthcare. We removed 139 instruments and other hospitals could achieve a similar reduction, because 134 of the removed instruments, we believe would also be removable from the international RSCs. We successfully eliminated three SITs and we are optimistic that other hospitals can achieve similar results. Extrapolating from our experience, implementing a three SITs reduction across all RSA could yield substantial environmental benefits.

## Conclusion

In orthopaedic surgery, reducing environmental impact should be a priority. Our study has shown that a reduction of 139 (55%) instruments and three SITs (30%) for RSA can be achieved. By using a supplementary tray, 19 additional instruments could be removed from the base set. This supplementary tray will only be needed for deviant sizes. The environmental impact of this reduction was 5.62 kg CO_2_-eq. per cycle/ procedure. SITs of RSA should undergo a thorough audit to determine if reduction and optimisation are possible. Following this initial rigorous audit, SITs should be re-evaluated each time changes or additions are made. Involvement of all stakeholders and the LEAN 5S method is essential for this, as this is not a straightforward task.

## Supplemental Material

sj-pdf-1-sel-10.1177_17585732251315424 - Supplemental material for Life cycle assessment and optimisation of surgical instrument trays for reverse shoulder arthroplasty Supplemental material, sj-pdf-1-sel-10.1177_17585732251315424 for Life cycle assessment and optimisation of surgical instrument trays for reverse shoulder arthroplasty by Isabella C Klarenbeek, Anne C van der Eijk, Esther RC Janssen, Freek Hollman, Paul C Willems and Okke Lambers Heerspink in Shoulder & Elbow
