# Supplementary material for: Life cycle assessment and optimisation of surgical instrument trays for reverse shoulder arthroplasty
Source: Shoulder Elbow. 2025 Jan 30;17(6):801–8. doi: 10.1177/17585732251315424 (PMC11780606; doi:10.1177/17585732251315424)
Supplement: sj-pdf-1-sel-10.1177_17585732251315424 - Supplemental material for Life cycle assessment and optimisation of surgical instrument trays for reverse shoulder arthroplasty [file sj-pdf-1-sel-10.1177_17585732251315424.pdf]

Life Cycle Analysis

Our hospital

	Emission factor	Units of measurement of EF	Quantity	Units of measurement of quantity	Total	Source
**Packaging**						
Two polypropylene wraps	1,63	kg CO2e/ kg	0,15	kg	0,25	Idemat 2024 V2
Transportation	0,088714534	kg CO2e/ km	0,09	tkm	0,01	Idemat 2024 V2
Paper inlay	0,955	kg CO2e/ kg	0,01	kg	0,01	Idemat 2024 V2
Paper protector	0,955	kg CO2e/ kg	0,03	kg	0,03	Idemat 2024 V2
Disposal polypropylene wraps	1,227009474	kg CO2e/ kg	0,15	kg	0,19	Idemat 2024 V2
Disposal paper	-1,60502	kg CO2e/ kg	0,04	kg	-0,06	Idemat 2024 V2
				per SIT:	**0,42**	
**Thermodesinfector**						
Electricity	0,0076406130732	kg CO2e/ kWh	3,08	Kw	0,02	Idemat 2024 V2
Cold water connection	0,000629156	kg CO2e/ kg	95,76	kg	0,06	Idemat 2024 V2
Hot water connection	0,000629156	kg CO2e/ kg	66,12	kg	0,04	Idemat 2024 V2
Demineralized Water Connection	0,000629156	kg CO2e/ kg	39,90	kg	0,03	Idemat 2024 V2
Soap	0,314		0,18	liter	0,05	Villota-Paz et al., 2023
Compressed air	0,007640613	kg CO2e/ kWh	0,02	kWh	0,00	Idemat 2024 V2
Inhouse steam	0,000629156	kg CO2e/ kg	17,10	kg	0,01	Idemat 2024 V2
Inhouse steam gas	4,801082481	kg CO2e/ m3	1,50	m3	7,22	Idemat 2024 V2

		Number of slots in machine:	12,00	Per slot:	**0,62**	
**Sterilisator**						
Electricity	0,007640613	kg CO2e/ kWh	3,36	Kw	0,03	Idemat 2024 V2
Cold water connection	0,000629156	kg CO2e/ kg	262,50	kg	0,17	Idemat 2024 V2
Compressed air	0,007640613	kg CO2e/ kWh	0,01	kWh	0,00	Idemat 2024 V2
Steam from steamconverter		steamconverter			12,90	
		Number of SITs in machine:	16,00	Per SIT:	**0,82**	
**Steamgenerator (Sterilizator)**						
Demiwateraansluiting	0,000629156	kg CO2e/ kg	20,00	kg	0,01	Idemat 2024 V2
Compressed air	0,007640613	kg CO2e/ kWh	0,03	kWh	0,00	Idemat 2024 V2
Inhouse steam	0,000629156	kg CO2e/ kg	29,00	kg	0,02	Idemat 2024 V2
Inhouse steam gas	4,801082481	kg CO2e/ m3	2,55	m3	12,25	Idemat 2024 V2
				Per hour:	**12,28**	
**Ultrasound machine**						
Water	0,000629156	kg CO2e/ kg	40,00	kg	0,03	Idemat 2024 V2
Electricity	0,007640613	kg CO2e/ kWh	0,07	kW	0,00	Idemat 2024 V2
		Number of nets in machine:	2	Per net:	**0,01**	
				**Total**	**1,87**	

				Calculation:
Opening of supplemental tray=	6,67%	One SIT=	1,87	F9+F20+F27+F39
		All SITs before change one cycle=	19,97	10*(F9+F27+F39)+12*F20
		Removed SITs one cycle=	5,62	3*D43
		% change one cycle	28,14	D45/D44*100
		Savings one year	524,34	D45*(100-100*B43)

Netherlands

NL	Electricity MJ	0,11402	Gas m3	4,801082481
		Idemat 2024 V2		Idemat 2024 V2

	Emission factor	Units of measurement of EF	Quantity	Units of measurement of quantity	Total	Source
**Packaging**						
Two polypropylene wraps	1,63	kg CO2e/ kg	0,15	kg	0,25	Idemat 2024 V2
Transportation	0,088714534	kg CO2e/ km	0,09	tkm	0,01	Idemat 2024 V2
Paper inlay	0,955	kg CO2e/ kg	0,01	kg	0,01	Idemat 2024 V2
Paper protector	0,955	kg CO2e/ kg	0,03	kg	0,03	Idemat 2024 V2
Disposal polypropylene wraps	1,227009474	kg CO2e/ kg	0,15	kg	0,19	Idemat 2024 V2
Disposal paper	-1,60502	kg CO2e/ kg	0,04	kg	-0,06	Idemat 2024 V2
				per SIT:	**0,42**	
**Thermodesinfector**						
Electricity	0,11402	kg CO2e/ kWh	11,08	MJ	1,26	Idemat 2024 V2
Cold water connection	0,000629156	kg CO2e/ kg	95,76	kg	0,06	Idemat 2024 V2
Hot water connection	0,000629156	kg CO2e/ kg	66,12	kg	0,04	Idemat 2024 V2
Demineralized Water Connection	0,000629156	kg CO2e/ kg	39,90	kg	0,03	Idemat 2024 V2
Soap	0,314		0,18	liter	0,05	Villota-Paz et al., 2023
Compressed air	0,11402	kg CO2e/ kWh	0,07	MJ	0,01	Idemat 2024 V2
Inhouse steam water	0,000629156	kg CO2e/ kg	17,10	kg	0,01	Idemat 2024 V2
Inhouse steam gas	4,801082481	kg CO2e/ m3	1,50	m3	7,22	Idemat 2024 V2
		Number of slots in machine:	12,00	Per slot:	**0,72**	

Sterilisator

Electricity	0,11402	kg CO2e/ kWh	3,36	Kw	0,38	Idemat 2024 V2
Cold water connection	0,000629156	kg CO2e/ kg	262,50	kg	0,17	Idemat 2024 V2
Compressed air	0,47340542	kg CO2e/ kWh	0,04	MJ	0,02	Idemat 2024 V2
Steam from steamconverter		steamconverter			12,95	
		Number of SITs in machine:	16,00	Per SIT:	**0,84**	

Steamgenerator (Sterilizator)

Demiwateraansluiting	0,000629156	kg CO2e/ kg	20,00	kg	0,01	Idemat 2024 V2
Compressed air	0,47340542	kg CO2e/ kWh	0,10	MJ	0,05	Idemat 2024 V2
Inhouse steam	0,000629156	kg CO2e/ kg	29,00	kg	0,02	Idemat 2024 V2
Inhouse steam gas	4,801082481	kg CO2e/ m3	2,55	kg	12,25	Idemat 2024 V2
				Per hour:	**12,33**	

Ultrasound machine

Water	0,000629156	kg CO2e/ kg	40,00	kg	0,03	Idemat 2024 V2
Electricity	0,11402	kg CO2e/ kWh	0,24	MJ	0,03	Idemat 2024 V2
		Number of SITs in machine:	2,00	Per SIT:	**0,03**	
				**Total**	**2,02**	

			Calculation:
One SIT=	2,02		F58+F69+F76+F88
Removed SITs one cycle=	6,05		3*D93
Potential yearly saving NL=	3000	18150	D96*D94

Europe

EU	Electricity MJ	0,08872	Gas kg	3,91071
	Idemat 2024 V2		Idemat 2024 V2	

Assumption density gas: 0.717 kg/m³

	Emission factor	Units of measurement of EF	Quantity	Units of measurement of quantity	Total	Source
**Packaging**						
Two polypropylene wraps	1,63	kg CO2e/ kg	0,15	kg	0,25	Idemat 2024 V2
Transportation	0,088714534	kg CO2e/ km	0,09	tkm	0,01	Idemat 2024 V2
Paper inlay	0,955	kg CO2e/ kg	0,01	kg	0,01	Idemat 2024 V2
Paper protector	0,955	kg CO2e/ kg	0,03	kg	0,03	Idemat 2024 V2
Disposal polypropylene wraps	1,227009474	kg CO2e/ kg	0,15	kg	0,19	Idemat 2024 V2
Disposal paper	-1,60502	kg CO2e/ kg	0,04	kg	-0,06	Idemat 2024 V2
				per SIT:	**0,42**	
**Thermodesinfector**						
Electricity	0,08872	kg CO2e/ kWh	11,08	MJ	0,98	Idemat 2024 V2
Cold water connection	0,000629156	kg CO2e/ kg	95,76	kg	0,06	Idemat 2024 V2
Hot water connection	0,000629156	kg CO2e/ kg	66,12	kg	0,04	Idemat 2024 V2
Demineralized Water Connection	0,000629156	kg CO2e/ kg	39,90	kg	0,03	Idemat 2024 V2
Soap	0,314		0,18	liter	0,05	Villota-Paz et al., 2023
Compressed air	0,08872	kg CO2e/ kWh	0,07	MJ	0,01	Idemat 2024 V2
Inhouse steam water	0,000629156	kg CO2e/ kg	17,10	kg	0,01	Idemat 2024 V2

Inhouse steam gas	3,91071	kg CO2e/ kg	1,08	kg	4,22	Idemat 2024 V2
		Number of slots in machine:	12,00	Per slot:	**0,45**	

Sterilisator

Electricity	0,08872	kg CO2e/ kWh	3,36	Kw	0,30	Idemat 2024 V2
Cold water connection	0,000629156	kg CO2e/ kg	262,50	kg	0,17	Idemat 2024 V2
Compressed air	0,47340542	kg CO2e/ kWh	0,04	MJ	0,02	Idemat 2024 V2
Steam from steamconverter		steamconverter			7,59	
		Number of slots in machine:	16,00	Per slot:	**0,50**	

Steamgenerator (Sterilizator)

Demiwateraansluiting	0,000629156	kg CO2e/ kg	20,00	kg	0,01	Idemat 2024 V2
Compressed air	0,47340542	kg CO2e/ kWh	0,10	MJ	0,05	Idemat 2024 V2
Inhouse steam	0,000629156	kg CO2e/ kg	29,00	kg	0,02	Idemat 2024 V2
Inhouse steam gas	3,91071	kg CO2e/ kg	1,83	kg	7,16	Idemat 2024 V2
				Per hour:	**7,23**	

Ultrasound machine

Water	0,000629156	kg CO2e/ kg	40,00	kg	0,03	Idemat 2024 V2
Electricity	0,08872	kg CO2e/ kWh	0,24	MJ	0,02	Idemat 2024 V2
		Number of SITs in machine:	2,00	Per SIT:	**0,02**	
				**Total**	**1,40**	

		Calculation:
One SIT=	1,40	F107+F118+F125+F137
Removed SITs one cycle=	4,20	3*D141

United Kingdom

UK	Electricity kg CO2e/ kWh	0,20705	Gas kg CO2e/ m3	2,04542
		DEFRA 2024		DEFRA 2024

United Kingdom	Emission factor	Units of measurement of EF	Quantity	Units of measurement of quantity	Total	Source
**Packaging**						
Two polypropylene wraps	1,63	kg CO2e/ kg	0,15	kg	0,25	Idemat 2024 V2
Transportation	0,088714534	kg CO2e/ km	0,09	tkm	0,01	Idemat 2024 V2
Paper inlay	0,955	kg CO2e/ kg	0,01	kg	0,01	Idemat 2024 V2
Paper protector	0,955	kg CO2e/ kg	0,03	kg	0,03	Idemat 2024 V2
Disposal polypropylene wraps	1,227009474	kg CO2e/ kg	0,15	kg	0,19	Idemat 2024 V2
Disposal paper	-1,60502	kg CO2e/ kg	0,04	kg	-0,06	Idemat 2024 V2
				per SIT:	**0,42**	
**Thermodesinfector**						
Electricity	0,20705	kg CO2e/ kWh	3,08	Kw	0,64	DEFRA 2024
Cold water connection	0,000629156	kg CO2e/ kg	95,76	kg	0,06	Idemat 2024 V2
Hot water connection	0,000629156	kg CO2e/ kg	66,12	kg	0,04	Idemat 2024 V2
Demineralized Water Connection	0,000629156	kg CO2e/ kg	39,90	kg	0,03	Idemat 2024 V2
Soap	0,314		0,18	liter	0,05	Villota-Paz et al., 2023
Compressed air	0,20705	kg CO2e/ kWh	0,02	kWh	0,00	DEFRA 2024
Inhouse steam water	0,000629156	kg CO2e/ kg	17,10	kg	0,01	Idemat 2024 V2
Inhouse steam gas	2,04542	kg CO2e/ m3	1,50	m3	3,08	Idemat 2024 V2
		Number of slots in machine:	12,00	Pet slot:	**0,33**	

Sterilisator

Electricity	0,20705	kg CO2e/ kWh	3,36	Kw	0,70	
Cold water connection	0,000629156	kg CO2e/ kg	262,50	kg	0,17	Idemat 2024 V2
Compressed air	0,47340542	kg CO2e/ kWh	0,01	kWh	0,01	Idemat 2024 V2
Steam from steamconverter		steamconverter			12,91	
		Number of SITs in machine:	16,00	Per SIT:	**0,86**	

Steamgenerator (Sterilizator)

Demiwateraansluiting	0,000629156	kg CO2e/ kg	20,00	kg	0,01	Idemat 2024 V2
Compressed air	0,47340542	kg CO2e/ kWh	0,03	kWh	0,01	DEFRA 2024
Inhouse steam	0,000629156	kg CO2e/ kg	29,00	kg	0,02	Idemat 2024 V2
Inhouse steam gas	4,801082481	kg CO2e/ m3	2,55	m3	12,25	Idemat 2024 V2
				Per hour:	**12,30**	

Ultrasound machine

Water	0,000629156	kg CO2e/ kg	40,00	kg	0,03	Idemat 2024 V2
Electricity	0,20705	kg CO2e/ kWh	0,07	kW	0,01	DEFRA 2024
		Number of SITs in machine:	2,00	Per SIT:	**0,02**	
				**Total**	**1,63**	

			Calculation:
One SIT=	1,63		F154+F165+F172+F184
Removed SITs one cycle=	4,88		3*D189
Potential yearly saving UK=	5000	24424	D192*D190

United States

US	Electricity MJ Idemat 2024 V2	0,14642	Gas =	54.81 thousand cubic feet kg co2 Per Unit of Volume or Mass
			Carbon factors provided by the U.S. Environmental Protection Agency, Inventory of U.S. Gronehouse Gas Emissions and Sinks: 1990-2022, Tables A-20, A-25, A-32, and A-226	

	Emission factor	Units of measurement of EF	Quantity	Units of measurement of quantity	Total	Source
**Packaging**						
Two polypropylene wraps	1,63	kg CO2e/ kg	0,15	kg	0,25	Idemat 2024 V2
Transportation	0,088714534	kg CO2e/ km	0,09	tkm	0,01	Idemat 2024 V2
Paper inlay	0,955	kg CO2e/ kg	0,01	kg	0,01	Idemat 2024 V2
Paper protector	0,955	kg CO2e/ kg	0,03	kg	0,03	Idemat 2024 V2
Disposal polypropylene wraps	1,227009474	kg CO2e/ kg	0,15	kg	0,19	Idemat 2024 V2
Disposal paper	-1,60502	kg CO2e/ kg	0,04	kg	-0,06	Idemat 2024 V2
				Per SIT:	**0,42**	
**Thermodesinfector**						
Electricity	0,14642	kg CO2e/ kWh	11,08	MJ	1,62	Idemat 2024 V2
Cold water connection	0,000629156	kg CO2e/ kg	95,76	kg	0,06	Idemat 2024 V2
Hot water connection	0,000629156	kg CO2e/ kg	66,12	kg	0,04	Idemat 2024 V2
Demineralized Water Connection	0,000629156	kg CO2e/ kg	39,90	kg	0,03	Idemat 2024 V2
Soap	0,314		0,18	liter	0,05	Villota-Paz et al., 2023
Compressed air	0,14642	kg CO2e/ kWh	0,07	MJ	0,01	
Inhouse steam water	0,000629156	kg CO2e/ kg	17,10	kg	0,01	Idemat 2024 V2

Inhouse steam gas	54,81	kg CO2e/ Volume (thousand cubic feet)	0,05	thousand cubic feet	2,91	U.S. EPA
		Number of slots in machine:	12,00	Per slot:	**0,39**	
**Sterilisator**						
Electricity	0,14642	kg CO2e/ kWh	3,36	Kw	0,49	
Cold water connection	0,000629156	kg CO2e/ kg	262,50	kg	0,17	Idemat 2024 V2
Compressed air	0,47340542	kg CO2e/ kWh	0,04	MJ	0,02	Idemat 2024 V2
Steam from steamconverter		steamconverter			5,27	
		Number of SITs in machine:	16,00	Per SIT:	**0,37**	
**Steamgenerator (Sterilizator)**						
Demiwateraansluiting	0,000629156	kg CO2e/ kg	20,00	kg	0,01	Idemat 2024 V2
Compressed air	0,47340542	kg CO2e/ kWh	0,10	MJ	0,05	Idemat 2024 V2
Inhouse steam	0,000629156	kg CO2e/ kg	29,00	kg	0,02	Idemat 2024 V2
Inhouse steam gas	54,81	kg CO2e/ Volume (thousand cubic feet)	0,09	thousand cubic feet	4,94	U.S. EPA
				Per hour:	**5,02**	
**Ultrasound machine**						
Water	0,000629156	kg CO2e/ kg	40,00	kg	0,03	Idemat 2024 V2
Electricity	0,14642	kg CO2e/ kWh	0,24	MJ	0,04	Idemat 2024 V2
		Number of SITs in machine:	2,00	Per SIT:	**0,03**	
				**Total**	**1,22**	
						Calculation:
		One SIT=	1,22			F203+F214+F221+F233

Removed SITs one cycle=	3,65		3*D237
Potential yearly saving US=	20000	73096	D240*D238
